# Calcinosis Universalis: An Atypical Presentation of Mi-2 Positive Juvenile Dermatomyositis - A Case-Based Review

**DOI:** 10.31138/mjr.291024.cua

**Published:** 2025-06-02

**Authors:** Rajat Kumar Sahu, Abhishek Gollarahalli Patel, Rajat Gupta, Kishan Majithiya, Urmila Dhakad

**Affiliations:** Department of Clinical Immunology and Rheumatology, King George’s Medical University, Lucknow, India

**Keywords:** dermatomyositis, calcinosis, Mi-2, case report

## Abstract

**Background:**

Juvenile Dermatomyositis (JDM) is a systemic autoimmune disease in children, characterised by skin and muscle inflammation, with incidence of 1.6 to 4 cases per million children annually. Calcinosis, affecting 20% to 70% of JDM patients, can lead to significant morbidity. The association of calcinosis with anti-Mi-2 antibodies is rare and complicates management.

**Case:**

We present a rare case of a 12-year-old girl diagnosed with JDM, extensive calcinosis, and positive anti-Mi-2 antibodies. The patient exhibited significant muscle weakness, skin manifestations, and painful calcinosis leading to contractures. Initial management included tofacitinib and intravenous pamidronate, resulting in no new calcinosis formation.

**Discussion:**

A comprehensive review of existing literature highlights the rarity of calcinosis in anti-Mi-2 positive patients. While traditional treatments have shown variable effectiveness, emerging therapies like JAK (Janus Kinase) inhibitors may offer new avenues for management. The literature underscores the need for personalised treatment strategies given the atypical presentations and outcomes.

**Conclusion:**

This case adds to the limited documentation of calcinosis in JDM with anti-Mi-2 antibodies, emphasising the need for increased awareness and research. Personalised treatment approaches are crucial, and future studies should focus on larger datasets and emerging therapeutic modalities to optimise management and improve patient outcomes.

## INTRODUCTION

Juvenile Dermatomyositis (JDM) is a systemic autoimmune disease in children, characterised by chronic inflammation of the skin and muscles. The reported incidence of JDM is between 1.6 and 4 cases per million children per year, with an estimated prevalence of 2.5 cases per 100,000 children.^1^ Calcinosis in the skin and subcutaneous tissue affects 20% to 70% of JDM patients^2^ and up to 20% of adults with DM.3 It can be painful and may lead to recurrent infections and local inflammation, causing significant discomfort and disability.^4^ Calcinosis is associated with anti-nuclear matrix protein 2 (anti-NXP-2), anti-Ro-52, and anti-melanoma differentiation-associated gene 5 (anti-MDA-5) antibodies in both adult and juvenile dermatomyositis. Despite severe skin involvement, transcriptional intermediary factor 1 gamma (anti-TIF-1- gamma) is negatively associated with calcinosis.^5^ The anti-PM-Scl antibody is associated with subcutaneous calcinosis in systemic sclerosis-inflammatory myopathy (IIM) overlap syndrome.^5^

Anti-Mi-2 antibodies are associated with typical cutaneous manifestations such as the shawl sign, V sign, and cuticular overgrowth but not with calcinosis.^6^ Anti-Mi-2 antibodies are associated with a good prognosis due to their steroid responsiveness. In contrast, calcinosis is an atypical feature in anti-Mi2 antibody-positive patients and is challenging to manage and poorly responsive to steroids. Calcinosis can appear as superficial papules or nodules, deeper nodules or tumours in the dermis or subcutaneous tissue, or diffuse deposits along the myofascial planes. If widespread, it can create a vast exoskeleton.^4^ Calcinosis Universalis is defined as the diffuse involvement of subcutaneous and fibrous structures of muscles and tendons whereas calcinosis circumscripta is limited to a joint or extremity.^4^

Here, we report a rare case of JDM with extensive calcinosis associated with anti-Mi-2 antibodies. The association of calcinosis with anti-Mi-2 antibodies is rare and not widely documented, making this case particularly noteworthy. We also conducted the literature review which provided valuable context by summarising existing knowledge on the prevalence, clinical presentation, and management of calcinosis in DM, particularly in the setting of anti-Mi-2 antibody positivity. By comparing this case to previously reported cases and treatment protocols, the review could guide clinicians in managing similar cases and contribute to the development of optimised treatment guidelines. An informed consent was obtained from the parent prior to the publication of any personal health information, ensuring adherence to ethical standards and patient confidentiality.

## CASE DESCRIPTION

**Figure 1.**
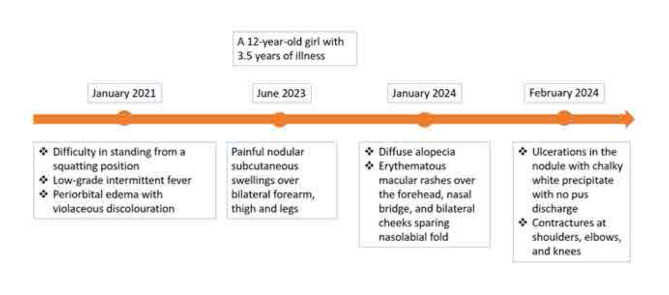
Timeline of events.

**Figure 2.**
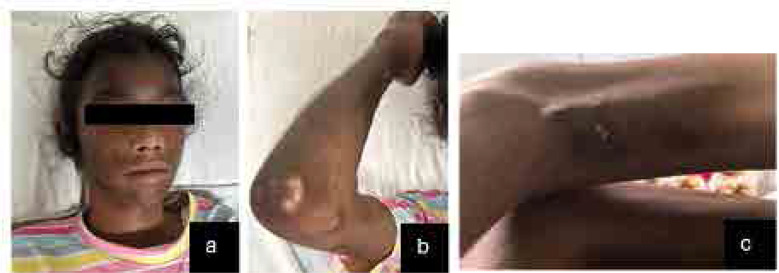
Examination findings of the case. **a)** Hyperpigmented macular rashes over the face and scalp sparing of nasolabial fold. **b)** Calcinosis over the right elbow joint. **c)** Calcinosis in the posterior aspect of thigh with chalky white precipitate.

A 12-year-old girl presented with periorbital oedema with violaceous discoloration for the last 3.5 years (**Figure 1**). She also experienced low-grade intermittent fever and progressive difficulty in standing up from a squatting position, which began insidiously and gradually worsened. Initially, she was able to perform daily activities independently, then with support, but for the past 6 months, she has been bedridden and requires assistance for daily tasks and self-care. Over the last one year, she developed painful nodular swellings over both thighs, legs, and forearms, which progressively increased in size, rendering her non-ambulatory for the past 6 months. Additionally, she had diffuse hair loss, along with erythematous macular rashes over her forehead, nasal bridge, and both cheeks, sparing the nasolabial folds (**Figure 2A**). These rashes were associated with photosensitivity and healed with hyperpigmentation over the last 6 months.

She also developed an ulcerated nodule on the posterolateral aspect of her left knee, without any pus discharge (**Figure 2C**), and developed contractures in both shoulders, elbows, and knees. There is no history of nasal intonation, regurgitation, dysphagia, dyspnoea, palpitations, abdominal pain, blood in the stool, decreased appetite, or contact with tuberculosis. She also denied oral ulcers, arthralgia, Raynaud’s phenomenon, oral or ocular dryness, or seizures.

On examination, the patient exhibited diffuse non-scarring alopecia and pallor. There was diffuse hyperpigmentation on the face, sparing the nasolabial folds, as well as on the forearms, arms, and legs (**Figure 2A**). Calcinosis was observed in the bilateral arms, forearms, thighs, and around the elbows and knees (**Figure 2B**). Notably, there was ulcerative calcinosis with chalky white discharge on the posterior aspect of the left knee, without any pus discharge (**Figure 2C**).

Her single breath count was 40. A complete MMT8 (Manual Muscle Testing) could not be performed due to contractures; however, significant weakness was noted, with muscle strength scored as 2/5 in the neck and deltoid muscles. Systemic examination was unremarkable.

**Figure 3.**
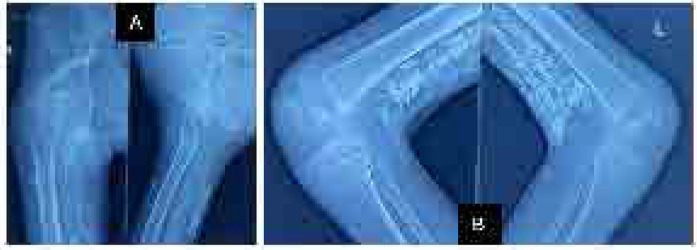

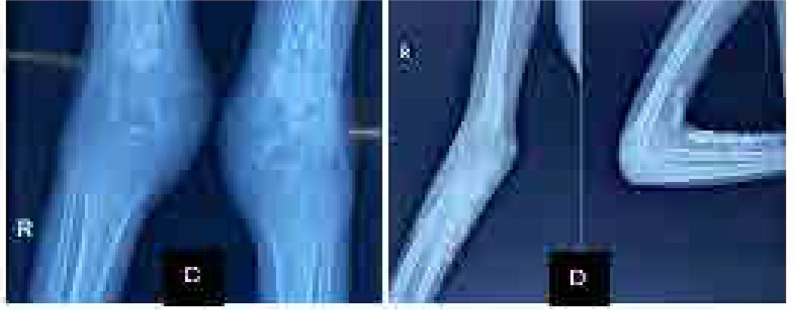
Conventional radiography of extremities. **A)** X-ray showing calcinosis around right hip joint and proximal thigh. **B)** X-ray of the knee showing calcinosis behind both knee joints and distal part of the posterior thigh. **C)** X-ray showing calcinosis in posterior aspect of the distal thigh. **D)** X-ray of bilateral elbow joint showing calcification in bilateral arms and forearms.

The investigations reveal anaemia with a microcytic hypochromic picture. Elevated liver enzymes (SGOT 117 IU/L, SGPT 78 IU/L) indicate possible muscle injury, as corroborated by a highly elevated creatinine phosphokinase (CPK) of 897 IU/L, and LDH levels of 1400 IU/L (**Table 1**). Positive antinuclear antibodies (ANA 4+) with a fine speckled pattern and positive Mi-2 antibodies (3+) were supportive of diagnosis of JDM. Conventional radiography revealed extensive calcinosis in the subcutaneous and intermuscular plane in both upper and lower extremities consistent with calcinosis universalis (**Figure 3**). The patient’s Childhood Myositis Assessment Scale (CMAS) score was 3, while the Cutaneous Dermatomyositis Disease Area and Severity Index (CDASI) showed an activity score of 1 and a damage score of 16, reflecting the significant impact of her condition. She presented with severe contractures around the knees due to the extensive calcinosis, limiting terminal knee extension of 30 degrees.

**Table 1.** Investigations of the case.

**Parameters**	**Patient’s value**	**Normal Range**
**Haemoglobin**	87 gm/L	(120–150gm/L)
**Peripheral smear**	Microcytic hypochromic RBC	
**Serum Glutamic Oxaloacetic Transaminase (SGOT**)	117 IU/L	(5–40 IU/L)
**Serum Glutamate Pyruvate Transaminase (SGPT)**	78 IU/L	(5–40 IU/L)
**Creatinine**	53 μ mol/L	(53– 97 μ mol/L)
**Total Protein**	89 g/L	(60–80 g/L)
**Albumin**	33.7 g/L	(35– 50 g/L)
**Creatinine Kinase (CK)**	897 IU/L	(22– 198 IU/L)
**Lactate dehydrogenase (LDH)**	1400 IU/L	(140–280 IU/L)
**Ferritin**	196 ng/ml	(13– 150 ng/ml)
**Anti-nuclear antigen (ANA)**	4+ nuclear fine speckled	
**Extractable Nuclear Antibody (ENA)**	Negative	
**Muscle-specific Antigen (MSA)**	3+ Mi 2a and Mi 2b	
**Urine Routine**	Normal	
**24-hour urine protein**	275 mg/day	(< 150 mg/day)
**HBsAg, HCV, HIV**	Non-reactive	
**2D Echocardiogram**	EF normal, mild TR, MPAP 33 mm hg	
**Mantoux test**	Negative	
**Chest X ray PA view**	Normal	

RBC: Red blood cell; SGOT: Serum Glutamic Oxaloacetic Transaminase; SGPT: Serum Glutamate Pyruvate Transaminase; CK: Creatinine Kinase; LDH: Lactate dehydrogenase; ANA: Anti-nuclear antigen; ENA: Extractable Nuclear Antibody; MSA: Muscle specific antigen; Mi 2: Protein in the nucleosome remodelling-deacetylase (NuRD); EF: Ejection fraction; TR: Tricuspid regurgitation; MPAP: Mean pulmonary arterial pressure.

A diagnosis of anti-Mi-2 associated juvenile dermatomyositis (JDM) with extensive calcinosis and contractures was established. Management included tapering dose of prednisolone starting with 1mg/kg, tofacitinib 4 mg twice daily according to her age and intravenous pamidronate at a dose of 1 mg/kg for three consecutive days. A long-term plan was initiated to continue pamidronate treatment monthly for six months. Additionally, she was referred for guided physiotherapy to address the contractures and improve her overall functional mobility. At 3 months follow up, she has no new calcinosis and no worsening of previously established calcinosis.

## REVIEW OF LITERATURE

To identify relevant case reports and studies on juvenile dermatomyositis (JDM) patients with calcinosis and anti-Mi2 antibodies, a comprehensive search strategy was conducted across PubMed, Google Scholar, and Web of Science. Search terms were carefully selected to include “Juvenile Dermatomyositis,” “Calcinosis,” “Anti-Mi2 Antibodies,” and “Case Report” or “Clinical Study.” In PubMed, the query (“calcinosis”[MeSH Terms] OR “calcinosis”[All Fields] OR “calcinoses”[All Fields]) AND (“dermatomyositis”[MeSH Terms] OR “dermatomyositis”[All Fields]) AND (“mi2”[All Fields] OR “mi 2”[All Fields] OR “anti mi2”[All Fields] OR “Anti-Mi-2”[All Fields] OR “anti mi2”[All Fields] OR “mi 2”[All Fields]) was used, applying filters for English language and article types. Scopus and Web of Science searches utilised similar queries to capture a broad range of scholarly articles and open-access journals.

**Figure 4.**
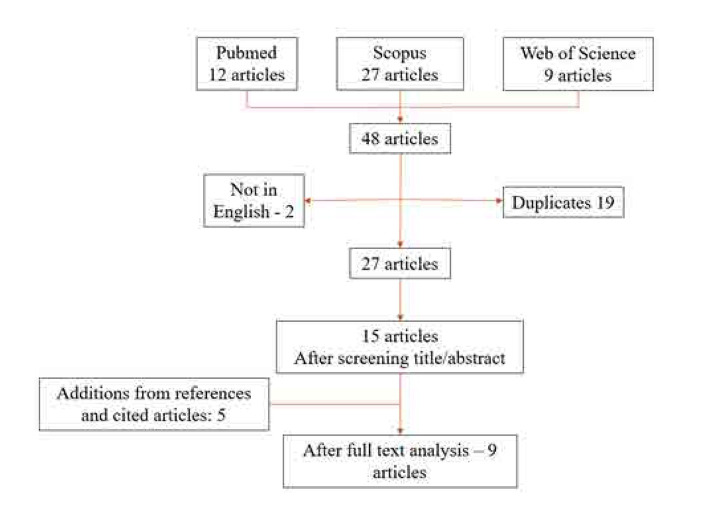
Flow chart of reviewed articles.

The inclusion criteria focused on studies or case reports specifically addressing JDM with calcinosis and anti-Mi2 antibodies, emphasising clinical details, treatment approaches, and outcomes. Articles not meeting these criteria or lacking full-text access were excluded. We retrieved a total of 48 articles and 9 articles were described after full text analysis (**Figure 4**).

After retrieving relevant articles, key data such as patient demographics, clinical features, and management strategies were extracted and reviewed. This systematic approach ensured a thorough examination of the literature, enabling a comprehensive analysis of the clinical characteristics and treatment outcomes associated with calcinosis in JDM patients with anti-Mi2 antibodies.

## DISCUSSION

Dermatomyositis (DM) is an idiopathic inflammatory myopathy characterised by distinctive clinical features, including muscle weakness, heliotrope rash, Gottron’s papules, poikiloderma, periungual telangiectasia, and alopecia.^7^ Calcinosis cutis is a well-known complication of dermatomyositis. It refers to aberrant calcium accumulation in the skin, subcutaneous tissue, fascia, and muscles.^8^ The mechanisms underlying calcinosis in dermatomyositis are multifaceted. One proposed mechanism is the release of calcium from mitochondria in muscle cells that are damaged due to myopathy.^9^ Macrophages, proinflammatory cytokines like IL 6, IL-1β, TNF α, and inhibition of calcium-regulating proteins have also been implicated.^10^ Other possible mechanisms are vascular hypoxia, recurrent trauma or damage, and bone marrow matrix protein dysregulation.^11^ Lower fetuin levels in the serum, high osteoprotegerin, and osteonectin also exacerbate calcinosis.^12^ In a single-centre experience, the most commonly detected antibodies were anti-NXP2 (30.2%), anti-TIF1γ (23.2%), and anti-MDA-5 (18.6%).^13^ Patients with anti-Mi-2 antibodies typically present with classic DM symptoms, including Gottron’s sign, heliotrope rashes, and rashes localised to the neck and upper back (often referred to as “V” and “shawl” rashes), as well as cuticular overgrowth.^14^ Calcinosis is an atypical manifestation of anti-Mi-2 antibodies. Untreated children with longer duration of disease correlate with calcinosis in JDM.^15^ Other risk factors are nailfold abnormalities at baseline, lipodystrophy, joint contracture, disease onset before 6 years of age.^16^

The table 2 provides an overview of various studies on calcinosis in dermatomyositis (DM), highlighting differences in patient demographics, study design, treatment approaches, and outcomes. The presence of anti-Mi2 antibodies associated with calcinosis was reported in a very small subset of patients across studies, indicating that while anti-Mi2 antibodies are generally linked with a favourable prognosis, their presence in conjunction with calcinosis may complicate disease course and management.

**Table 2.** Demographics, clinical features, treatment and outcomes of anti-Mi-2 positive dermatomyositis patients with calcinosis.

**Author**	**Valenzuela et al.^19^**	**Ibarra et al.^20^**	**Fernandez et al.^6^**	**Fornaro et al.^29^**	**Jiang et al.^8^**	**Monseau et al.^30^**	**Liang et al.^31^**	**Linan-Barroso et al.^17^**	**Vishwanath et al.^18^**	**Our study**
**Country**	USA	USA	USA	Italy	China	France	China	Spain	India	India
**Study design**	Cross-sectional	Prospective cohort	Longitudinal cohort	Retrospective observational Cohort	Retrospective Cohort	Retrospective	Cross-sectional	Case Report	Case report	Case report
**Year**	2014	2016	2019	2020	2020	2020	2020	2022	2024	2024
**Number of cases**	126 (17 Mi-2 +ve)	10 juvenile (1 Mi-2 +ve)	533 (58 Mi-2 +ve)	437 (22 Mi-2 +ve)	627 (31 Mi-2 +ve)	119 (64 Mi-2 +ve)	357 (40 Mi-2 +ve)	1	1	1 juvenile
**Calcinosis**	14	10	58	10	35	7	8	1	1	1
**Anti-Mi 2 positive calcinosis**	3	1	5	1	1	1	1	1	1	1
**Calcinosis location**	Extremities	Extremities, chest	Extremities	Extremities, abdomen	Trunk, extremities	Extremities, abdomen	Extremities	Extensive lumbar belt calcinosis	Arm, thigh, chest, iliac crest	Extremities
**Mean age of onset (in years)**	45.9	14	48	56.5	35	55.5	48	50	38	12
**Gender (% females)**	78%	80%	68%	75%	71%	60%	68%	F	F	F
**Disease duration before calcinosis (in years)**	6.9	8.6 months	4.3	5.8	3.5	3	7 months	3 months	12	2.5
**Treatment approach**	NA	IVIG, MTX, MMF, Cyclosporin	IVIG, MMF, MTX, AZA	IVIG, RTX, MTX, pamidronate	IVIG, pamidronate, prednisolone	NA	IVIG MTX, MMF HCQS	Prednisolone, MTX, IVIG, alendronate	Prednisolone, alendronate, MMF	Prednisolone, tofacitinib, pamidronate
**Complications**	Ulcer	No	No	No	Ulcerated calcinosis	Skin ulceration	No	Calcinosis increasing	Calcinosis non resolving	Skin ulceration
**Treatment response**	Not available	Average decrease in calcinosis volume by volume by CT 1.32cm3	Calcinosis resistant to the treatment	Not available	Resistant to treatment. Had monocyclic, polycyclic, chronic calcinosis	Not available	72% complete Remission, 22% Partial Remission	Even non-responding to Rituximab, diltiazem, sodium thiosulfate	No response	No worsening

All the available literature on the topic postdates 2014, with the largest study on myositis, conducted by Fernandez et al., reporting the highest number of cases, i.e. five patients.^6^ Calcinosis was primarily confined to the extremities, although two case reports documented involvement of the trunk or abdomen.^17,18^ The time between disease onset and calcinosis varied significantly, ranging from as short as 3 months to as long as 6.9 years, as noted in a case report by Valenzuela et al.^19^ In Ibarra et al.’s study of juvenile dermatomyositis, the average disease duration before the appearance of calcinosis was 8.6 months,^20^ while in our patient, calcinosis developed after 2.5 years.

The treatment approaches and responses to therapy also reveal a wide spectrum of effectiveness. For instance, Ibarra et al. employed a combination of IVIG, methotrexate, and other immunosuppressants with a notable reduction in calcinosis volume,^20^ whereas Jiang et al. and Fernandez et al. observed no response to multiple treatment modalities, including Rituximab and sodium thiosulfate.^6,8^ This disparity in treatment response highlights the challenge in managing calcinosis, especially when it presents as an atypical feature of anti-Mi2 antibody-positive DM. The varied treatment strategies, from IVIG and corticosteroids to more novel approaches like diltiazem, reflect ongoing efforts to address this condition.^17^

Standardised recommendations for the management of calcinosis are lacking, making it a clinical difficulty. According to the Childhood Arthritis and Rheumatology Research Alliance (CARRA), more experienced physicians treat calcinosis with bisphosphonates, calcium channel blockers, and topical sodium thiosulphate in decreasing order of preference.^21^ According to various reports in JDM patients, calcinosis has clearly improved or even vanished with bisphosphonate therapy.^22–24^

Type I interferon plays a role in the pathophysiology of dermatomyositis by promoting the expression of proinflammatory cytokines. Additionally, the JAK-STAT pathway may be involved in controlling the release of calcium stores from mitochondria, a process that may be crucial for calcification in the disease. Therefore, JAK inhibition may be a desirable treatment for DM that is complicated by calcifications. There are 2 case reports showing good results of tofacitinib in DM with calcinosis along with ILD.^25–27^ None of the patients developed new calcinosis or any worsening over 28 weeks of follow-up.^27^ According to the MyoIN registry, 38% of patients had calcinosis and tofacitinib use had a complete and /or partial response in 23/37 (64.8%), 30/35 (85.7%), and 29/30 (96.6%) patients at 3, 6, and 12 months, respectively. There was also a reduction in glucocorticoid dose after 12 months.^28^

Our patient is being managed with 1mg/kg prednisolone with serial tapering, tofacitinib 4mg BD according to the weight and IV pamidronate 1mg/kg for 3 consecutive days in every 3 months. She has no new calcinosis and no worsening of previously established calcinosis.

Moreover, the impact of calcinosis on patients’ quality of life is evident, with complications such as skin ulceration and increasing calcinosis being reported. The effectiveness of treatments ranges from significant reduction in calcinosis volume to resistance in some cases, emphasising the need for personalised treatment strategies and further research to optimise management. These findings underscore the complexity of treating calcinosis in DM and the necessity for continued exploration of both conventional and innovative therapeutic options to improve patient outcomes. The strengths of our study include a comprehensive analysis of a rare case of juvenile dermatomyositis (JDM) associated with extensive calcinosis and anti-Mi-2 antibodies, contributing valuable insights to the existing literature. The detailed case description, including clinical features, treatment approaches, and outcomes enhances understanding of the complexities involved in managing this condition. Additionally, the literature review contextualises the case by summarising previous studies, elucidating the association between calcinosis and specific antibody profiles, and highlighting the challenges in treatment efficacy. This thorough approach allows for a better understanding of the clinical presentation and management strategies in atypical cases, potentially guiding clinicians in similar scenarios.

However, the study has certain limitations. The review relies on existing literature, which may not encompass all recent developments or diverse treatment modalities, particularly the data that were not open access or in other languages, potentially affecting the comprehensiveness of the recommendations. The lack of standardised treatment protocols for calcinosis further complicates the formulation of a unified management strategy. Moreover, the treatment outcomes presented are based on a single case, limiting the ability to draw definitive conclusions about the effectiveness of the management strategies employed.

## CONCLUSION

This study presents a unique case of JDM associated with extensive calcinosis and the presence of anti-Mi-2 antibodies, a combination rarely documented in the literature. The detailed clinical presentation, diagnostic criteria, and management strategies underscore the complexity of treating calcinosis in JDM, highlighting the challenges clinicians face with this atypical manifestation. While anti-Mi-2 antibodies are generally linked to a favourable prognosis, their association with calcinosis complicates management and can lead to significant morbidity. This case contributes valuable insights to the existing knowledge base, emphasising the need for personalised treatment approaches and further research to establish effective management protocols. Ultimately, it calls for increased awareness among healthcare providers regarding the potential for calcinosis in JDM and encourages continued exploration of optimal therapeutic strategies to enhance patient outcomes.

## FUTURE PERSPECTIVES

Future research should focus on multi-centre studies to collect larger datasets that can elucidate the relationship between anti-Mi-2 antibodies and calcinosis in JDM. Investigating the underlying mechanisms that contribute to calcinosis in this context will enhance our understanding and could lead to the identification of novel therapeutic targets. Additionally, prospective clinical trials evaluating the efficacy of various treatment modalities, including emerging therapies like JAK inhibitors and bisphosphonates, will be essential in establishing evidence-based guidelines for managing calcinosis in JDM. Finally, further exploration into patient-reported outcomes will provide valuable insights into the quality-of-life impacts associated with calcinosis and inform holistic treatment approaches.

## AUTHOR CONTRIBUTIONS

The conception and design of the study – RKS, AGP, RG, KM, UD; acquisition of data, analysis and interpretation of data – RKS, AGP, RG, KM, UD.

Drafting the article – RKS, AGP, KM, UD; Revising it critically for important intellectual content – RKS, AGP, KM, UD.

Final approval of the version to be submitted – All authors.

Agreement to be accountable for all aspects of the work in ensuring that questions related to the accuracy or integrity of any part of the work are appropriately investigated and resolved – All authors.

## CONFLICT OF INTEREST

None of the authors declare any conflict of interest.

## FUNDING

No funds were received for this study.

## INFORMED CONSENT

An informed consent was obtained from the parent prior to the publication of any personal health information, ensuring adherence to ethical standards and patient confidentiality.

## DISCLAIMER

No part of this manuscript is copied or published elsewhere. No part of this study has been presented in any national or international conferences.
